# A comprehensive framework for multi-class prostate cancer classification using biparametric MRI: a multi-center retrospective study

**DOI:** 10.1186/s13244-026-02367-5

**Published:** 2026-08-17

**Authors:** Bowen Zheng, Yuling Yang, Maierhaba Maheremu, Liuru Bao, Ling Zhang, Genggeng Qin, Zhongjian Liao, Shidong Lv, Jie Tao, Yang Xiang, Qiang Wei

**Affiliations:** 1 Department of Diagnostic Imaging, Nanfang Hospital, Southern Medical University, Guangzhou, China; 2 Department of Medical Imaging, Zhongshan Hospital of Traditional Chinese Medicine, Guangdong, China; 3 Department of Urology, Nanfang Hospital, Southern Medical University, Guangzhou, China; 4 Department of Urology, Guangdong Provincial People’s Hospital, Guangdong Academy of Medical Sciences, Southern Medical University, Guangzhou, China; 5 Medical Imaging Department of Ganzhou People’s Hospital, Ganzhou, China; 6 School of Automation, Guangdong University of Technology, Guangzhou, China; 7 Guangdong Provincial Key Laboratory of Intelligent Decision and Cooperative Control, Guangzhou, China; 8 Guangdong-Hong Kong Joint Laboratory for Intelligent Decision and Cooperative Control, Guangzhou, China; 9 Department of Plastic Surgery, The First Affiliated Hospital of Ningbo University, Ningbo, China

**Keywords:** Prostate cancer, Risk stratification, bpMRI, Deep learning, Machine learning

## Abstract

**Objectives:**

Accurate pre-biopsy or preoperative diagnosis and risk stratification of prostate cancer remain challenging. This study aimed to develop an automated framework for prostate and suspicious lesion segmentation, feature extraction, and multiclass classification of prostate cancer.

**Materials and methods:**

This study enrolled 2543 patients with suspected clinically significant prostate cancer (csPCa). An nnUNet was used for prostate segmentation, followed by suspected lesion identification and feature extraction. Unimodal and multimodal deep learning models were trained to classify patients into non-csPCa (benign/ISUP 1), mid-risk csPCa (ISUP 2–3), and high-risk csPCa (ISUP 4–5). Subsequently, we extracted prostate radiomics, lesion radiomics, and learned features from the unimodal DL model. Radiomics features, learned features from the unimodal deep learning model, and clinical variables were integrated to develop machine-learning classifiers. The best-performing model was compared with conventional clinical variables and evaluated across subgroups defined by PSA levels and PI-RADS 4.

**Results:**

The nnUNet achieved scores of 0.971 in internal validation and 0.934 in external testing. The optimal machine learning model, which integrated all features, achieved a macro-AUC of 0.87 [0.84–0.90] across both internal and external evaluations. Further comparisons indicate it outperforms traditional metrics such as total PSA, PSAD, and PI-RADS. Additionally, it exhibits strong robustness in most subgroup analyses, consistently surpassing conventional variables.

**Conclusion:**

We developed an automated bpMRI-based framework that segments the prostate and suspicious lesions and enables multiclass risk stratification of prostate cancer by integrating imaging and clinical features. The proposed approach demonstrated strong performance and robustness, outperforming conventional clinical variables.

**Key Points:**

**Question** accurate pre-biopsy stratification of prostate cancer is essential for clinical decision-making. Current studies predominantly focus on binary classification of csPCa, and multi-class classification remains challenging.

**Findings** the comprehensive framework achieved an area under the curve of 0.87 in both internal and external tests, exceeding clinical variables and demonstrating robustness.

**Critical relevance statement** this study presents a robust multi-class classification framework based on biparametric MRI and machine learning to improve pre-biopsy prostate cancer stratification, outperform conventional metrics, and provide a valuable tool for risk stratification and clinical decision-making.

**Graphical Abstract:**

**Figure:**
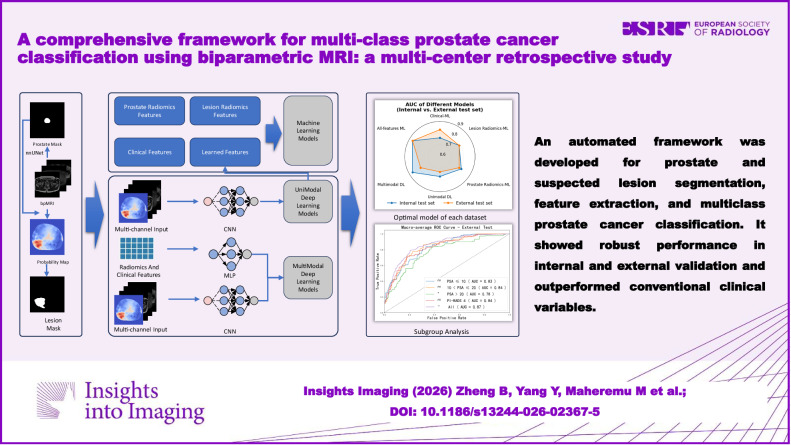

## Introduction

Prostate cancer (PCa) is the second most common cancer and the fifth leading cause of cancer-related deaths globally. It is the most frequently diagnosed cancer in 118 countries and the leading cause of cancer deaths in 58 countries [1]. Prostate-specific antigen (PSA) is the primary test for suspected PCa. While PSA aids in PCa screening, it can also lead to unnecessary biopsies and complications [2]. In patients with elevated PSA, magnetic resonance imaging (MRI) is widely used for further evaluation because it can detect and localize clinically significant PCa (csPCa). The Prostate Imaging-Reporting and Data System (PI-RADS) was developed to standardize MRI interpretation and enhance PCa diagnosis [3]. However, even with PI-RADS, the consistency of MRI interpretation still needs improvement [4, 5]. Other diagnostic tools include PSA density, prostate-specific membrane antigen positron emission tomography (PSMA-PET), and blood and urine biomarkers [6]. Using a combination of these methods may improve diagnostic accuracy for prostate cancer.

The binary diagnosis of PCa or csPCa offers limited clinical guidance. Accurate stratification of prostate cancer is crucial because of its high heterogeneity, which can range from slow progression or inactivity to aggressive, fatal disease [7]. Prostate cancer with International Society of Urological Pathology (ISUP) grade 2 or higher could be defined as csPCa, indicating a potential for morbidity or death. Additionally, prostate cancer with ISUP grades 4 or 5 is categorized as high-risk. The optimal treatment for high-risk csPCa remains uncertain, but these patients are at higher risk of undertreatment and recurrence [6]. The binary classification of non-csPCa vs csPCa may result in either overtreatment or undertreatment. Proper stratification of prostate cancer could identify the most effective treatment by aligning treatment intensity with the disease’s level of aggressiveness [8, 9].

In response to the complex data and precision-medicine requirements, Artificial Intelligence (AI) is increasingly applied in prostate cancer research. Numerous studies aim to improve the accuracy and efficiency of AI in diagnosing prostate cancer, with advancements evolving from radiomics [10, 11] to deep learning (DL) [12, 13], from single modality [14] to multimodal features [15, 16], and from manual to automated ROI delineation [17, 18]. However, most studies focus on binary classification of PCa or csPCa, while multi-level stratification remains limited; existing multi-class approaches often rely on cascade frameworks, which may propagate errors.

This study aims to develop and validate an automated bpMRI-based framework for multi-class classification of prostate cancer. Using DL and machine learning with imaging and clinical variables, the framework segments the prostate and suspicious lesions and extracts features. It stratifies patients into non-csPCa (benign or ISUP 1), mid-risk csPCa (ISUP 2–3), and high-risk csPCa (ISUP 4–5), supporting improved risk stratification and clinical decision-making.

## Materials and methods

### Population

**Fig. 1:**
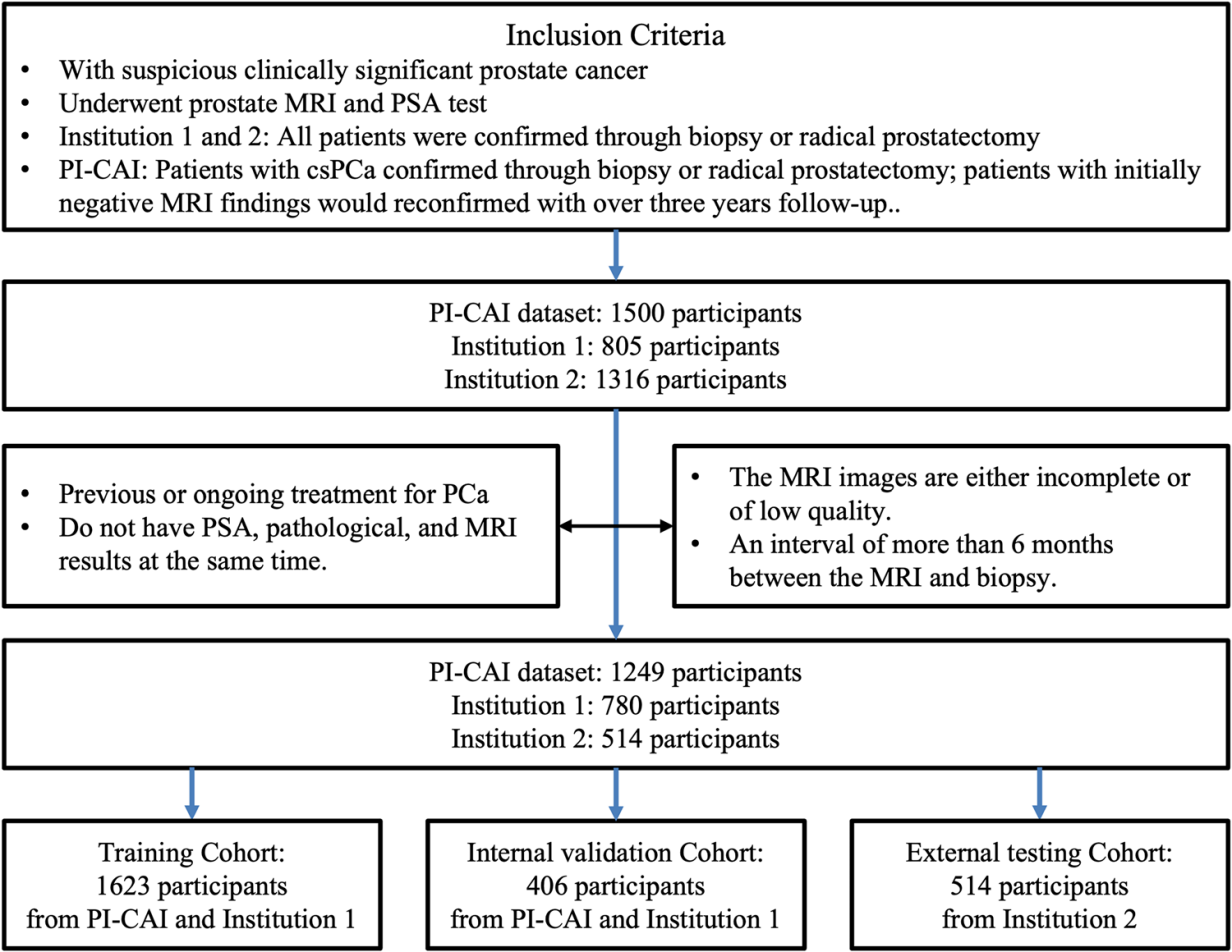
The flow diagram displays the initial number of participants, exclusion criteria, and final participants

This multi-center retrospective study utilizes one public dataset, Prostate Imaging: Cancer AI (PI-CAI) [19], and data from two local institutions. The PI-CAI dataset comprises data from three Dutch centers. It includes patients suspected of having csPCa who have no prior treatment history. Two local institutions included patients suspected of having csPCa from January 2015 to December 2022 in Ganzhou, Jiangxi Province, and Guangzhou, Guangdong Province, China. Exclusion criteria were (1) previous or ongoing treatment for PCa; (2) poor-quality MRI; and (3) missing prostate biopsy results or an interval of more than 6 months between the MRI and biopsy. Figure 1 shows the data inclusion process.

Histopathological reference standards were obtained from systematic biopsy (SysBx) combined with TRUS-guided biopsy or radical prostatectomy (RP) at two local institutions. For the PI-CAI dataset, MRI-positive cases were confirmed by SysBx, MRI-guided biopsy (MRBx), combined SysBx and MRBx, or RP, while MRI-negative cases were reconfirmed through follow-up exceeding 3 years. Clinical variables, including age, total PSA (tPSA), PSA density (PSAD), free PSA (fPSA), and the free-to-total PSA (F/T PSA), were collected. BpMRI was acquired using multiple scanners, with detailed parameters provided in Table S1. This retrospective study was approved by the Institutional Review Board (NFEC-2022-224), with informed consent waived.

### Prostate and suspicious lesion segmentation

**Fig. 2:**
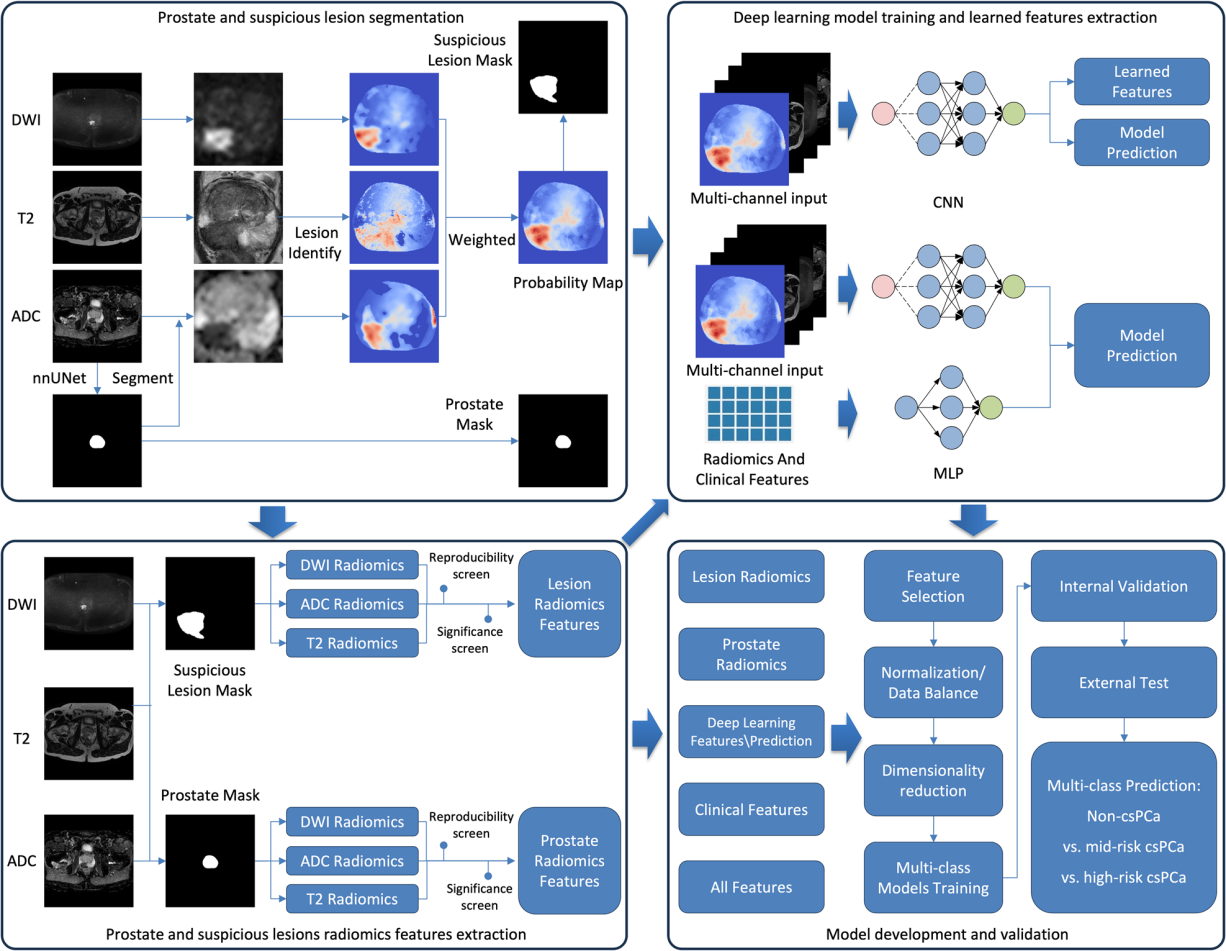
Illustration of the framework. An nnUNet is used to segment the entire prostate, while a feature-extraction module identifies suspicious lesions and produces a probability map. This probability map is then binarized to create a mask for the suspicious lesions. The bpMRI, along with the prostate mask, probability map, and lesion mask, will be used to extract features for DL, prostate radiomics, and lesion radiomics. After selecting features, normalizing data, and balancing classes, multiple machine learning models will be trained to classify non-csPCa, mid-risk csPCa, and high-risk csPCa

Figure 2 provides an overview of the proposed framework, which consists of segmentation, feature extraction, and model development. The first step is to segment the prostate and any suspicious lesions. An nnUNet was trained on the PI-CAI dataset for whole-prostate segmentation using T2-weighted imaging (T2WI) [20]. This adaptive segmentation framework automatically configures a U-Net-based pipeline by analyzing training samples. Its automated approach has proven effective, delivering exceptional performance across various datasets. The ground truth for segmentation in PI-CAI was obtained from the dataset, whereas for institutions 1 and 2, it was manually delineated by three radiologists with over three years of experience and reviewed by two radiologists with more than ten years of experience.

Most prostate cancers are focal lesions, so further lesion segmentation facilitates the extraction of more discriminative features. We used a knowledge-based feature-extraction module from our previous work to segment the suspicious lesion [21]. This module detects symmetric signal-intensity differences across the entire prostate segmented by nnUNet from T2WI, ADC, and diffusion weighted imaging (DWI), mimicking methods used by radiologists in clinical practice. The weighted sum of differences across these three sequences will be used to create a feature map that quantifies signal abnormalities and highlights suspicious regions. The feature map will be binarized using a threshold of 0.95 to create the suspicious lesions mask. This threshold was chosen based on an observational study of 20 cases per ISUP grade, in which various thresholds were evaluated, with 0.95 proving optimal.

### Radiomics features extraction

After preprocessing bpMRI (Supplementary Methods S1), radiomics features were extracted from the ROI segmented by nnUNet. To ensure intra-observer reproducibility, each case was segmented twice using the same nnU-Net model, and radiomics features were extracted from both segmentations. Features with an intraclass correlation coefficient above 0.9 were deemed highly reproducible and retained for further analysis. Radiomics features were extracted using Pyradiomics (v3.0.1) from the prostate and suspicious lesion masks aligned to each bpMRI sequence [22]. The least absolute shrinkage and selection operator would then be applied for significance screening after assessing reproducibility. Finally, radiomics features from T2WI, ADC, and DWI were combined to create the final dataset for the prostate and suspicious lesions.

### Development and validation of DL models and learned feature extraction

The DL models included bpMRI DL models as well as multimodal DL models that integrate clinical features, radiomics features, and bpMRI. For bpMRI DL models, the feature maps generated by the prior knowledge–guided prostate feature extraction module, together with the T2WI, DWI, and ADC images, were combined to form a four-channel input for training convolutional neural networks (CNNs), including ResNet [23] and DenseNet [24]. In multimodal models, CNNs extract bpMRI features, while a multi-layer perceptron extracts clinical and radiomics features. These features would be concatenated to produce the final prediction. The models were trained for three-class classification (non-csPCa, mid-risk csPCa, and high-risk csPCa). Training details were described in Supplementary Methods S2.

After training, the predicted probabilities and features from the fully connected layer of bpMRI DL models would be extracted. Since the number of learned features exceeded the size of our dataset, we applied dimensionality reduction methods, including Principal Component Analysis (PCA), Kernel PCA, and t-distributed Stochastic Neighbor Embedding, to reduce variance, improve stability, and enhance computational efficiency. The dimensionality-reduced features from the three methods, along with the predicted probabilities, would be used as learned features for further analysis. The feature extraction process is shown in Fig. 2.

### Development and validation of machine learning models

Four datasets were generated for machine learning model development: lesion radiomics, prostate radiomics, clinical features, and a comprehensive dataset that includes the above three datasets and a learned features dataset. For each dataset, twelve machine learning models were employed to classify non-csPCa, mid-risk csPCa, and high-risk csPCa. These models included logistic regression (LR), CatBoost Classifier, and linear discriminant analysis (LDA), among others. Together, they represent a diverse set of linear, probabilistic, instance-based, and ensemble approaches, enabling a systematic evaluation of suitability and robustness.

Training data were *Z*-score normalized and balanced using the synthetic minority oversampling technique. Models were trained on the internal cohort and validated on both internal and external cohorts. The best-performing model was compared with PSA, PSAD, F/T PSA, and PI-RADS in the external cohort to evaluate clinical utility. Subgroup analyses were performed in the external cohort stratified by PSA levels (< 10, 10–20, and > 20 ng/mL) and PI-RADS 4. PI-RADS 3 was excluded because most cases were pathologically confirmed as non-csPCa.

### Statistical analysis

Segmentation performance was evaluated using the Dice coefficient. Model performance was assessed using AUC, accuracy, sensitivity, precision, and F1 score. Bootstrapping (5000 iterations) was used to compare multi-class and binary models, and Decision Curve Analysis (DCA) was used to evaluate clinical benefit relative to standard methods. Statistical significance was defined as *p* < 0.05. All analyses were performed in Python (v3.11).

## Results

### Population characteristics

A total of 2543 participants with suspected clinically significant prostate cancer (csPCa) were enrolled after exclusion, including 1249 participants from the PI-CAI dataset, 780 participants from institution 1, and 514 participants from institution 2. Table 1 displays the basic, clinical, and pathological information. Data from the PI-CAI dataset and Institution 1 were used as internal cohorts, divided into a training set and an internal validation set at an 8:2 ratio. Data from Institution 2 were used as an external test cohort (training:internal validation:external test = 1623:406:514).

**Table 1:** The basic, clinical, and pathologic features of participants

Characteristic	PI-CAI	Institution 1	Institution 2	Total
Participants	1249	780	514	2543
Age (years)	65.05 ± 7.08	70.50 ± 7.84	66.94 ± 8.82	67.10 ± 8.05
PSA (ng/mL)	11.03 ± 11.88	65.33 ± 236.41	39.07 ± 191.08	32.10 ± 154.59
Volume (mL)	66.36 ± 37.03	54.68 ± 34.79	56.96 ± 34.47	60.83 ± 36.23
PSAD	0.21 ± 0.34	2.01 ± 8.93	0.99 ± 6.40	0.88 ± 5.63
ISUP grade				
0	816	433	357	1606
1	218	33	21	272
2	126	45	32	203
3	51	65	26	142
4	20	108	33	161
5	18	96	45	159
PI-RADS				
1	-	-	-	-
2	-	-	15	15
3	-	-	195	195
4	-	-	208	208
5	-	-	96	96

*PSA* prostate-specific antigen, *PSAD* PSA density, *PCa* prostate cancer, *ISUP* International Society of Urological Pathology, *PI-RADS* prostate imaging reporting and data system, *PI-CAI* prostate imaging: cancer artificial intelligence

### Performance of nnUNet in prostate segmentation

**Fig. 3:**
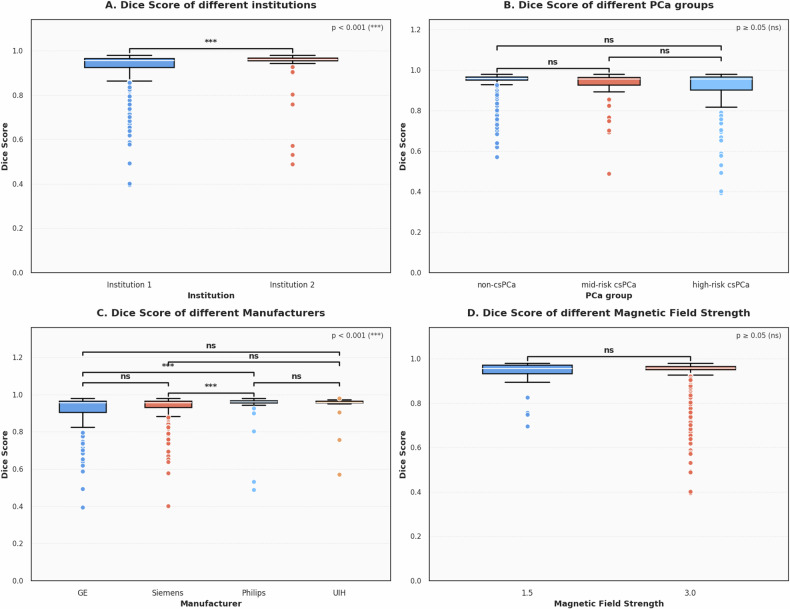
The performance of nnUNet prostate segmentation across institutions and imaging settings. **A** Segmentation performance across different institutions. **B** Segmentation performance across non-csPCa, mid-risk csPCa, and high-risk csPCa. **C** Segmentation performance across MRI scanner manufacturers. **D** Segmentation performance across different magnetic field strengths. ns, *p* > 0.05; ^*^p < 0.05; ^**^p < 0.01

The nnUNet model achieved a Dice score of 0.971 in internal validation on the PI-CAI dataset and 0.934 in external testing across institutions 1 and 2, with scores of 0.923 and 0.955, respectively. As shown in Fig. 3, the Dice coefficient decreases with increasing malignancy in the external test (non-csPCa: 0.945; mid-risk csPCa: 0.926; high-risk csPCa: 0.911). Segmentation using Philips MRI machines achieved the highest Dice coefficient (0.956), and segmentation at 3.0-T field strength outperformed that at 1.5T (0.935 vs 0.928).

### Comparative analysis of model performance across diverse datasets

The final comprehensive framework was a CatBoost classifier that integrated lesion radiomics, prostate radiomics, clinical variables, and learned features, including predicted probabilities and features from the fully connected layer of DenseNet264. It achieved micro-AUCs of 0.93 (95% CI: 0.91–0.95) and 0.92 (95% CI: 0.90–0.94) in internal and external cohorts, respectively, with a consistent macro-AUC of 0.87 (95% CI: 0.84–0.90). It outperformed single-modality machine learning and DL models across AUC, accuracy, precision, sensitivity, and F1 (Table 2). Although the optimal multimodal DenseNet-169 showed comparable internal performance, its external performance declined markedly. Furthermore, it outperformed other models in both multi-class and binary classification, with most ROC comparisons statistically significant (Fig. S1). The detailed results are shown in Table S2. Importantly, the proposed framework demonstrated excellent robustness, with no significant differences between internal and external cohorts in micro-ROC, macro-ROC, or binary classification (*p* > 0.05), and no significant decline in accuracy, precision, sensitivity, or F1 score (Fig. S2).

**Table 2:** Overall multi-class classification performance for each dataset during internal validation and external testing

	Dataset	Accuracy	Precision	Sensitivity	F1	Macro-average AUC	*p*-value^*^
Internal validation (*n* = 406)	Clinical	0.78	0.70	0.78	0.72	0.76 (0.72–0.80)	< 0.001
	Lesion radiomics	0.76	0.71	0.76	0.71	0.79 (0.75–0.83)	0.004
	Prostate radiomics	0.78	0.74	0.78	0.74	0.81 (0.77–0.84)	0.006
	Unimodal DL	0.76	0.72	0.76	0.73	0.77 (0.72–0.82)	0.002
	Multi-modality DL	0.70	0.28	0.89	0.43	0.87 (0.84–0.90)	0.91
	All^#^	0.79	0.76	0.79	0.76	0.87 (0.84–0.90)	-
External test (*n* = 514)	Clinical	0.76	0.66	0.76	0.70	0.83 (0.80–0.87)	0.104
	Lesion radiomics	0.74	0.69	0.74	0.69	0.79 (0.74–0.83)	< 0.001
	Prostate radiomics	0.76	0.70	0.76	0.70	0.79 (0.75–0.84)	0.006
	Unimodal DL	0.70	0.68	0.70	0.69	0.73 (0.69–0.78)	< 0.001
	Multi-modality DL	0.60	0.17	0.54	0.26	0.79 (0.75–0.83)	0.006
	All^#^	0.78	0.74	0.78	0.75	0.87 (0.84–0.90)	-

^*^ The *p*-value was calculated using bootstrap with 5000 iterations to compare the AUC of the model trained on all data vs each individual dataset

^#^ The model was trained on a comprehensive dataset that includes clinical features, lesion radiomics, prostate radiomics, and DL features

### Impact of segmentation accuracy on multi-class classification analysis

**Fig. 4:**
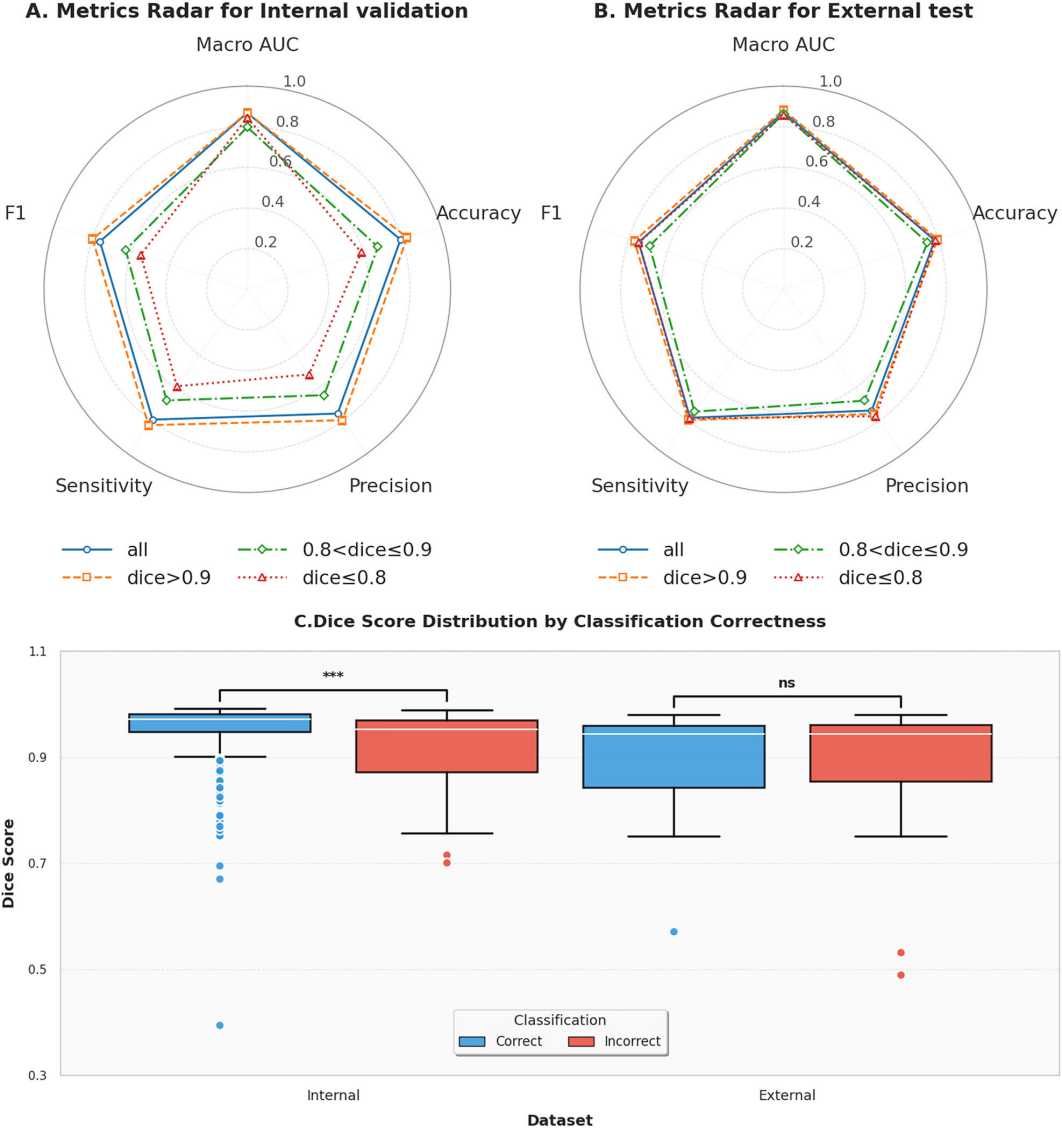
Impact of prostate segmentation accuracy on multi-class classification performance. **A**, **B** Radar plots summarizing multi-class classification performance under different prostate segmentation quality levels for the internal validation cohort (**A**) and the external test cohort (**B**). **C** Boxplots comparing prostate segmentation Dice scores between correctly and incorrectly classified cases in the internal and external datasets. ns, *p* > 0.05; ^*^p < 0.05; ^**^p < 0.01

Figure 4 shows the association between nnUNet-derived prostate segmentation quality and PCa multi-class classification performance. In the internal validation cohort, higher Dice scores were associated with better classification performance across all evaluated metrics. In addition, Dice scores were significantly higher in correctly classified cases than in incorrectly classified cases (*p* < 0.001). In the external test cohort, classification performance was similar across Dice-based subgroups, and no significant difference in Dice scores was observed between correctly and incorrectly classified cases.

### The comprehensive framework outperforms conventional clinical variables

**Fig. 5:**
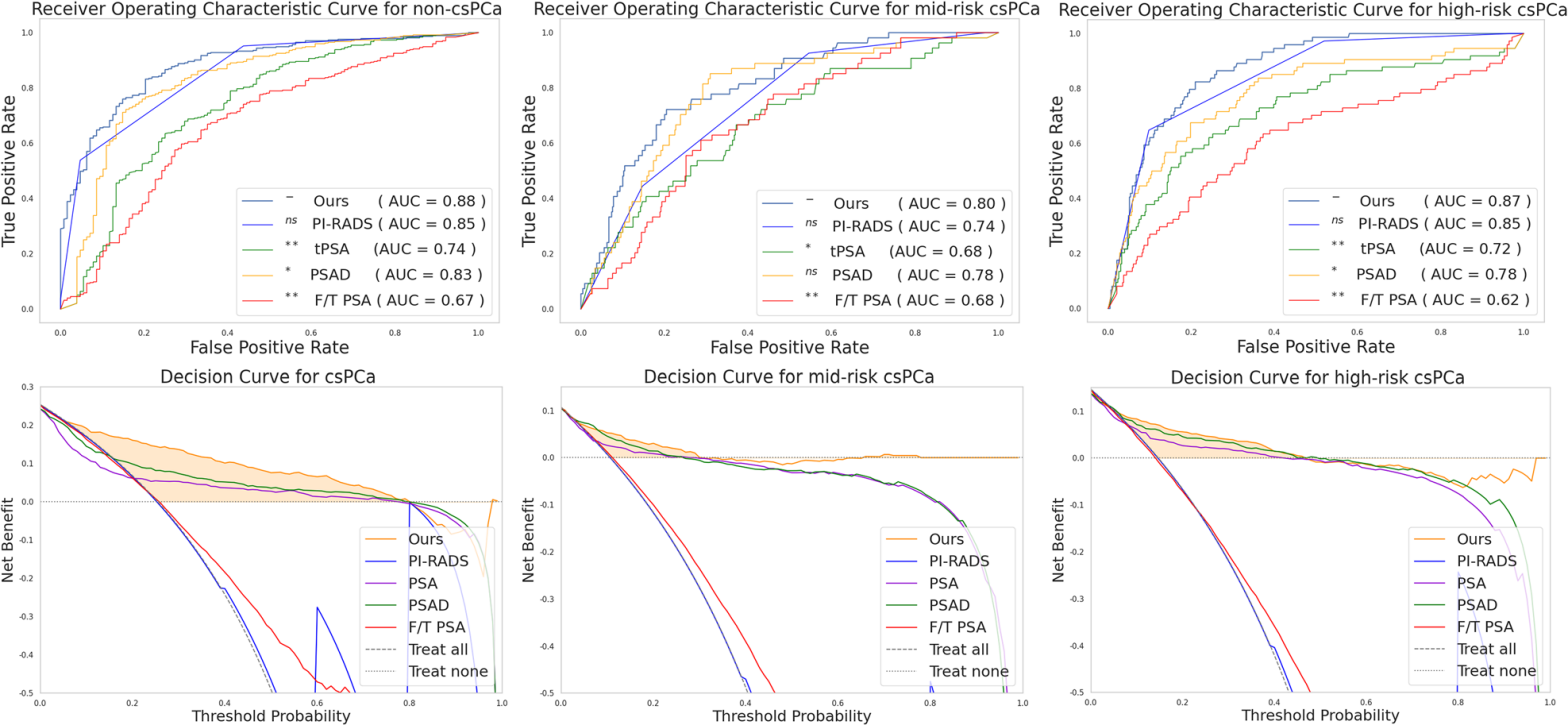
Comparison of the ROC curve and decision curve for our comprehensive framework, PI-RADS, tPSA, PSAD, and F/T PSA in external testing. The non-csPCa classification was converted into csPCa in the decision curve. The *p*-value is calculated using bootstrapping with 5000 iterations. ns, *p* > 0.05; ^*^p < 0.05; ^**^p < 0.01

As shown in Fig. 5, the comprehensive framework outperformed traditional clinical variables in prostate cancer diagnosis. Multi-class predictions were converted to binary classifications for comparison with clinical variables (Table 3). Our comprehensive framework achieved AUCs of 0.88 (95% confidence interval (CI): 0.85–0.91) for non-csPCa, 0.80 (95% CI: 0.75–0.86) for mid-risk csPCa, and 0.87 (95% CI: 0.84–0.91) for high-risk csPCa, exceeding the performance of tPSA, PSAD, F/T PSA, and PI-RADS. Performance differences were statistically significant for all comparisons except PI-RADS, which achieved AUCs of 0.85, 0.74, and 0.85 for the three categories, respectively.

**Table 3:** Comparison of performance for diagnosing non-csPCa, mid-risk csPCa, and high-risk csPCa of our comprehensive framework with tPSA, PSAD, F/T PSA, and PI-RADS in the external cohort

Task	Method	AUC (95% CI)	Optimal threshold	Sensitivity	Specificity	*p*-value^*^
Non-csPCa (benign/ISUP 1, *n* = 378)	Ours	0.88 (0.85–0.91)	0.629	0.832	0.797	-
	tPSA	0.74 (0.69–0.79)	0.385	0.635	0.750	< 0.001
	PSAD	0.83 (0.78–0.87)	0.566	0.723	0.844	0.040
	F/T PSA	0.67 (0.62–0.73)	0.318	0.669	0.648	< 0.001
	PI-RADS	0.85 (0.82–0.88)	0.514	0.952	0.563	0.360
Mid-risk csPCa (ISUP 2–3, *n* = 58)	Ours	0.80 (0.75–0.86)	0.517	0.722	0.795	-
	tPSA	0.68 (0.60–0.75)	0.295	0.741	0.555	0.016
	PSAD	0.78 (0.72–0.84)	0.542	0.852	0.690	0.592
	F/T PSA	0.68 (0.62–0.76)	0.324	0.611	0.713	< 0.001
	PI-RADS	0.74 (0.68–0.80)	0.380	0.926	0.454	0.220
High-risk csPCa (ISUP 4–5, *n* = 78)	Ours	0.87 (0.83–0.91)	0.615	0.824	0.790	-
	tPSA	0.72 (0.66–0.79)	0.383	0.635	0.748	< 0.001
	PSAD	0.78 (0.72–0.84)	0.478	0.676	0.802	0.030
	F/T PSA	0.62 (0.54–0.69)	0.262	0.635	0.627	< 0.001
	PI-RADS	0.85 (0.81–0.89)	0.551	0.649	0.902	0.380

^*^ The *p*-value was calculated using bootstrap with 5000 iterations to compare the AUC of the model trained on all data vs each dataset

*AUC* area under the curve, *F/T PSA* the ratio of free to total PSA, *ISUP* International Society of Urological Pathology, *PSA* prostate-specific antigen, *PSAD* prostate-specific antigen density, *csPCa* clinically significant prostate cancer, *PI-RADS* prostate imaging reporting and data system

To improve clarity, non-csPCa cases were reclassified as csPCa for DCA. The proposed framework demonstrated a higher net benefit than conventional clinical variables across csPCa, mid-risk csPCa, and high-risk csPCa, whereas PI-RADS showed limited net benefit, likely because all patients in the external cohort have undergone biopsy or RP, resulting in most PI-RADS scores being higher than 3.

### The comprehensive automatic framework demonstrating robust performance in subgroup analyses

The proposed framework demonstrated consistently strong performance across subgroups (Fig. S3), with no significant differences from overall performance except for reduced accuracy in the PSA > 20 ng/mL subgroup. The macro-AUC was 0.87 overall and for high-risk csPCa, compared with 0.78 and 0.77, respectively, in the PSA > 20 ng/mL subgroup. Moreover, as shown in Fig. S4, the framework outperformed traditional clinical variables in binary classification across all subgroups, except for high-risk csPCa prediction in patients with PSA levels of 10–20 ng/mL.

## Discussion

In this study, we developed an automated bpMRI-based framework that segments the prostate and suspicious lesions, extracts radiomics and learned features, and classifies patients into non-csPCa, csPCa, and high-risk csPCa using imaging and clinical variables. This framework achieved robust performance in both internal and external tests and outperformed conventional clinical variables such as PSA, PSAD, F/T PSA, and PI-RADS score.

**Fig. 6:**
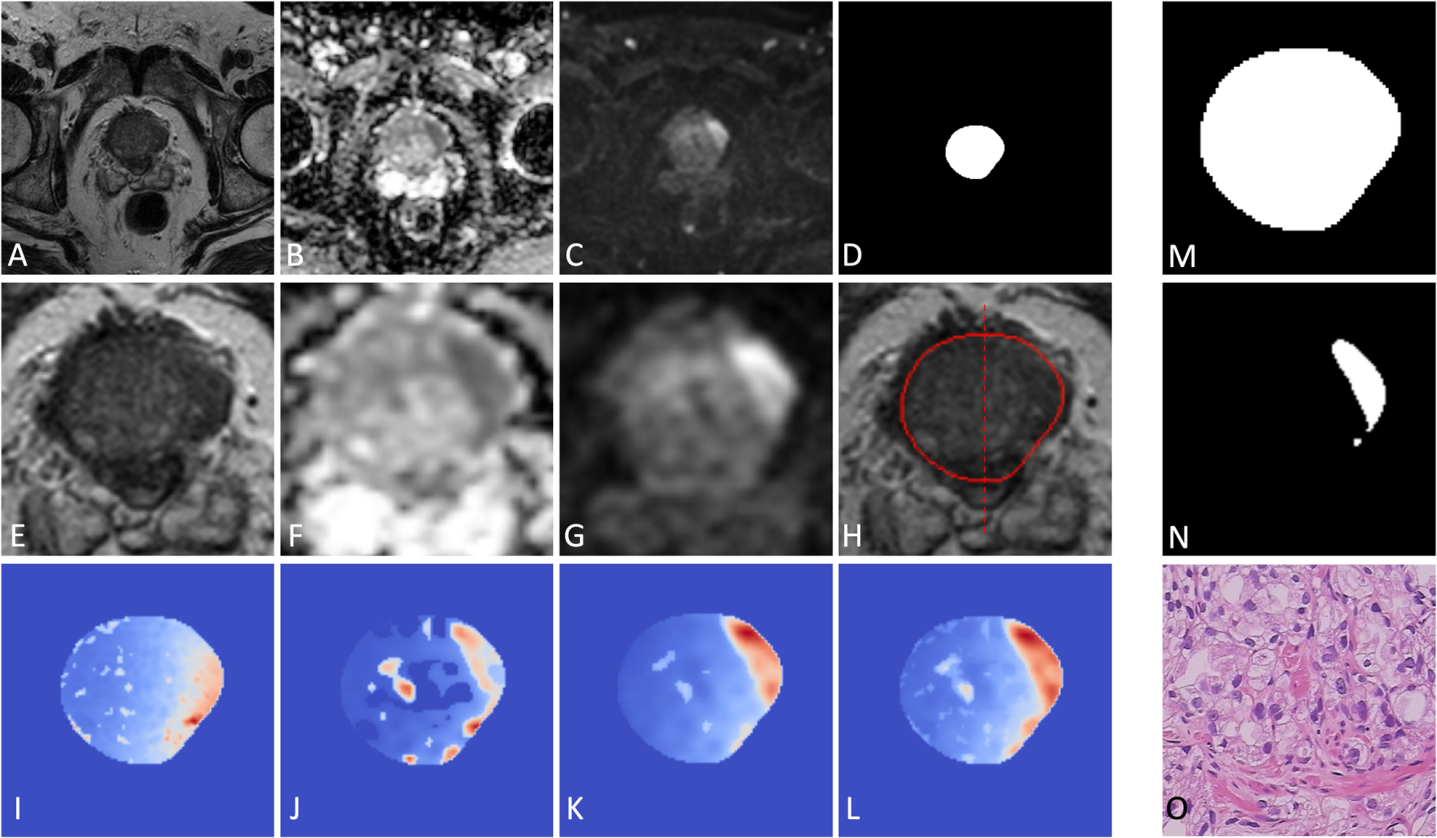
Example illustrating the overall workflow of our proposed method. **A**–**C** Original input of T2WI, ADC, and DWI. **D** Prostate mask generated by nnU-Net based on T2WI. **E**–**G** T2WI, ADC, and DWI images cropped based on the mask. **H** Schematic diagram of abnormal areas calculated within the prostate mask. **I**–**K** Feature map of suspicious lesion areas on T2WI, ADC, and DWI. **L** Feature map of suspicious lesion areas after weighting different sequences. **M** The cropped prostate mask generated by nnU-Net for further analysis. **N** The binary suspicious lesion mask generated from the weighted feature map. **O** HE-stained pathological image of this case

Accurate lesion identification is critical for model performance. Our comprehensive framework utilized nnUNet and a feature extraction method to segment the prostate and suspicious lesions, ensuring accurate feature extraction at both global and local levels. Previous studies typically use manual lesion segmentation. Liechti et al examined the inter-reader agreement in manual prostate cancer segmentation and found a moderate level of consistency in MRI segmentations [25]. While DL has greatly improved prostate segmentation, accurately segmenting prostate lesions remains challenging. For instance, Schelb et al developed a DL model to detect and classify csPCa, achieving Dice coefficients of 0.89 for prostate segmentation and 0.35 for lesion segmentation [26, 27]. Our approach identifies abnormal signal asymmetry within the prostate, mimicking radiologists’ practice. By integrating with nnUNet, we enhance the effectiveness of feature extraction at both global and local levels. As illustrated in Fig. 6, a T2WI-derived prostate mask is first generated to crop the biparametric MRI sequences. Within this mask, abnormal signals at symmetrical regions across sequences are extracted, weighted, and binarized to define the suspicious lesion mask. Ultimately, features extracted from both the prostate and lesion masks are utilized for multi-class lesion classification. Our model predicted this case as high-risk csPCa, which is consistent with the pathological finding of ISUP grade 4.

Prostate cancer stratification is essential for treatment selection. Treatment options for intermediate-risk patients consist of active surveillance, partial gland ablation, and RP [9]. For high-risk patients, there is no universally recommended treatment; instead, a multimodal approach is essential to optimize outcomes [8]. Currently, there are numerous pretreatment stratification tools, including the European Association of Urology (EAU) [6], the Cancer of the Prostate Risk Assessment (CAPRA) score [28], Cambridge Prognostic Groups (CPG) risk group systems [29], and the Memorial Sloan Kettering Cancer Center (MSKCC). PSA and PSAD are commonly used biomarkers, with thresholds of < 10 and > 20 ng/mL often defining low- and high-risk disease; however, PSA lacks specificity and may be elevated in benign conditions or remain normal in aggressive cancers [30]. Although PSA-based metrics contribute to risk assessment, pathological evaluation remains the cornerstone of prostate cancer stratification [6, 29].

Our method demonstrated improved accuracy compared with conventional variables for prostate cancer diagnosis and stratification. With MRI now recommended for biopsy decision-making, its increased use has improved csPCa detection while substantially reducing unnecessary biopsies [31–33]; however, it also poses challenges in maintaining diagnostic quality under growing workloads [34]. AI-assisted analysis offers a promising solution to enhance accuracy and efficiency. Most previous studies focus on the binary csPCa classification. For instance, Cai et al reported a DL model for csPCa detection with an AUC of 0.86 in external testing [35]. Zhao et al developed a DL approach for PCa and csPCa, which achieved AUCs of 0.82–0.87 and 0.84–0.87 across multiple external cohorts [36]. In contrast, we further classified patients into three categories according to the EAU guideline criteria. Our comprehensive framework achieved an AUC of 0.87 in external testing and maintained robust performance across subgroups, except for a decline in the PSA > 20 subgroup. This decline likely results from intratumoral heterogeneity in this group and differences in data distribution between the training and external testing sets. Future inclusion of larger multicenter datasets may help mitigate this limitation and further improve model generalizability. By integrating radiomics, learned features, and clinical features, our framework enables accurate multi-class prediction, facilitating preoperative risk stratification and more precise treatment decisions. In addition to machine learning models, we also explored unimodal and multimodal DL models. Although the multimodal DL models exhibited limited generalizability and imbalanced performance, their improved performance compared with image-only DL models demonstrates their potential.

This study has several limitations. Although we included a public dataset from three institutions and two local cohorts, the sample size should be further expanded to address inter-dataset heterogeneity. The PI-CAI dataset lacked MRI and pathology reports, and one local institution did not provide PI-RADS scores, limiting the assessment of pathological features (e.g., lymph node metastasis) and thereby restricting the study to shared labels such as ISUP. The absence of PI-RADS scores also confined subgroup analyses to the external test cohort. Additionally, our feature extraction method serves as an alternative for inadequate lesion segmentation in current DL approaches. Compared with DL-based approaches, this method does not directly segment prostate lesions. Instead, it progressively narrows the ROI based on the prostate ROI to approximate suspicious lesion regions. Therefore, we did not assess this method’s performance in segmenting suspicious lesions. Finally, our framework performs only patient-level classification. Future improvements in lesion segmentation are expected to enable more accurate lesion-level analysis.

In conclusion, we developed an automated bpMRI-based framework that identifies the prostate and suspicious lesions, extracts multimodal features, and stratifies patients into non-csPCa, mid-risk csPCa, and high-risk csPCa categories. The framework outperformed conventional clinical variables and PI-RADS in both binary and multi-class classification, supporting improved risk stratification and clinical decision-making while potentially reducing under- and over-treatment.

## Supplementary information

ELECTRONIC SUPPLEMENTARY MATERIAL

## Data Availability

The public dataset analyzed during the study is available at https://pi-cai.grand-challenge.org/DATA/. The private datasets generated or analyzed during the study are not publicly available due to privacy concerns but are available from the corresponding author on reasonable request.

## Abbreviations

AI: Artificial intelligence
AUC: Area under the curve
CI: Confidence interval
CNNs: Convolutional neural networks
csPCa: Clinically significant prostate cancer
DCA: Decision curve analysis
DL: Deep learning
DWI: Diffusion weighted imaging
EAU: European Association of Urology
F/T PSA: The ratio of free to total PSA
ISUP: International Society of Urological Pathology
MRBx: MRI-guided biopsy
MRI: Magnetic resonance imaging
PCa: Prostate cancer
PI-CAI: Prostate imaging: cancer artificial intelligence
PI-RADS: Prostate imaging reporting and data system
PSA: Prostate-specific antigen
PSAD: Prostate-specific antigen density
RP: Radical prostatectomy
SysBx: Systematic biopsy
T2WI: T2 weighted imaging
