# Supplementary material for: A comprehensive framework for multi-class prostate cancer classification using biparametric MRI: a multi-center retrospective study
Source: Insights Imaging. 2026 Aug 17;17:203. doi: 10.1186/s13244-026-02367-5 (PMC13481968; doi:10.1186/s13244-026-02367-5)

A Comprehensive Framework for Multi-class Prostate Cancer Classification Using Biparametric MRI: A Multi-center Retrospective Study

ELECTRONIC SUPPLEMENTARY MATERIAL

Insights Imaging (2026) Zheng B, Yang Y, Maheremu M et al.

Supplement Methods S1

Preprocessing of bpMRI

Because the bpMRI were acquired on different machines, all images were resampled for consistency. Image intensities were standardized to 0-255, and spatial resolution was set to $0.5 \times 0.5 \times 3.0$ mm. DWI and ADC images were also resampled to align with T2WI and match the prostate and suspicious masks.

Supplement Methods S2

Training of Deep Learning Models

Multiple CNN models were trained, including ResNet (ResNet50, ResNet101, ResNet152, ResNet200) and DenseNet (DenseNet121, DenseNet169, DenseNet201, DenseNet264). The models were trained for a three-class classification task (non-csPCa, mid-risk csPCa, and high-risk csPCa) with a batch size of 8 for 300 epochs. Data augmentation included random cropping, intensity shifting, affine transformation, scaling, denoising, and contrast enhancement. The initial learning rate was set to 0.0001 and optimized using a cosine annealing schedule with a warm-up phase during the first ten epochs. AdamW optimization and focal loss were used, with mixed-precision training and an exponential moving average applied to enhance training efficiency and model robustness.

Supplement Table S1 The MRI scanners and parameters for each dataset.

Institution	n	Manufactory	Modality	Field Strength	Echo Time	Repetition Time	Slice Thickness
PI-CAI dataset	830	Siemens Skyra	T2WI, DWI	3.0 T	-	-	-
	60	Siemens TrioTim	T2WI, DWI	3.0 T	-	-	-
	69	Siemens Prisma	T2WI, DWI	3.0 T	-	-	-
	17	Siemens Aera	T2WI, DWI	1.5 T	-	-	-
	13	Siemens Avanto	T2WI, DWI	1.5 T	-	-	-
	208	Philips Ingenia	T2WI, DWI		-	-	-
	52	Philips Achieva	T2WI, DWI	3.0 T	-	-	-
Institution 1	283	GE MR750w	T2WI	3.0 T	102 - 137	3243-10254	3.0-5.0
			DWI	3.0 T	70-83	2564 -7001	3.5-5.0
	229	Siemens Verio	T2WI	3.0 T	96 - 101	3600-7450	3.0-4.5
			DWI	3.0 T	93	6500-10500	3.0-5.0
	211	Siemens Skyra	T2WI	3.0 T	78 - 101	4800-7730	3.0-4.0
			DWI	3.0 T	61	4700-7000	3.0-4.0
	57	Siemens Aera	T2WI	1.5 T	107 - 120	8000-9520	3.0-5.0
			DWI	1.5 T	70	5900-7500	3.0-5.0
Institution 2	406	Philips Achieva	T2WI	3.0 T	90 - 110	2900-5000	3.0
			DWI	3.0 T	50 - 79	1798-6000	3.0-4.0
	43	UIH uMR 780	T2WI	3.0 T	127-130	2932-5874	3.0
			DWI	3.0 T	76	2974-6675	3.0-3.5
	38	Philips Ingenia	T2WI	3.0 T	110	4175-5610	3.0
			DWI	3.0 T	82 - 84	3882-5000	3.0
	27	Siemens Vida	T2WI	3.0 T	112	7800-9750	3.0
			DWI	3.0 T	75 - 79	5000-6300	3.0

Supplement Table S2 Overall performance of all models for multi-class prostate cancer classification (non-csPCa, mid-risk csPCa, high-risk csPCa) using various datasets.

Dataset	Model	Accuracy	AUC	Recall	Precision	F1
Clinical + Lesion Radiomics+ Prostate Radiomics+ Learned features	*CatBoost Classifier	0.79	0.87	0.79	0.76	0.76
	Linear Discriminant Analysis	0.79	0.85	0.79	0.76	0.77
	Extreme Gradient Boosting	0.79	0.86	0.79	0.75	0.76
	Logistic Regression	0.77	0.85	0.77	0.71	0.73
	Light Gradient Boosting Machine	0.78	0.87	0.78	0.75	0.75
	Gradient Boosting Classifier	0.79	0.86	0.79	0.75	0.76
	Extra Trees Classifier	0.79	0.86	0.79	0.74	0.74
	Naive Bayes	0.78	0.82	0.78	0.73	0.75
	Random Forest Classifier	0.80	0.86	0.80	0.75	0.75
	Quadratic Discriminant Analysis	0.69	0.83	0.69	0.75	0.71
	Ada Boost Classifier	0.77	0.80	0.77	0.73	0.74
	K Neighbors Classifier	0.75	0.75	0.75	0.71	0.72
Clinical	*Linear Discriminant Analysis	0.78	0.76	0.78	0.70	0.72
	Logistic Regression	0.76	0.75	0.76	0.67	0.70
	Naive Bayes	0.78	0.76	0.78	0.71	0.73
	Quadratic Discriminant Analysis	0.77	0.75	0.77	0.72	0.73
	Gradient Boosting Classifier	0.76	0.75	0.76	0.71	0.73
	Random Forest Classifier	0.73	0.74	0.73	0.70	0.71
	CatBoost Classifier	0.74	0.74	0.74	0.70	0.71
	Light Gradient Boosting Machine	0.74	0.74	0.74	0.72	0.73
	Extra Trees Classifier	0.73	0.75	0.73	0.70	0.71
	Extreme Gradient Boosting	0.75	0.73	0.75	0.72	0.73
	K Neighbors Classifier	0.74	0.70	0.74	0.70	0.71
	Ada Boost Classifier	0.77	0.69	0.77	0.69	0.71
Lesion Radiomics	Logistic Regression	0.76	0.76	0.76	0.71	0.69
	*CatBoost Classifier	0.76	0.79	0.76	0.71	0.71
	Linear Discriminant Analysis	0.75	0.76	0.75	0.70	0.71
	Extreme Gradient Boosting	0.76	0.77	0.76	0.70	0.71
	Extra Trees Classifier	0.75	0.78	0.75	0.66	0.67
	Light Gradient Boosting Machine	0.75	0.77	0.75	0.70	0.71
	Gradient Boosting Classifier	0.75	0.75	0.75	0.69	0.69
	Quadratic Discriminant Analysis	0.63	0.76	0.63	0.70	0.65
	Random Forest Classifier	0.75	0.78	0.75	0.65	0.66
	Naive Bayes	0.75	0.76	0.75	0.72	0.73
	K Neighbors Classifier	0.75	0.74	0.75	0.69	0.69
	Ada Boost Classifier	0.73	0.69	0.73	0.67	0.69
Prostate Radiomics	Logistic Regression	0.75	0.80	0.75	0.68	0.68
	Linear Discriminant Analysis	0.77	0.80	0.77	0.73	0.74
	*CatBoost Classifier	0.78	0.81	0.78	0.74	0.74
	Light Gradient Boosting Machine	0.77	0.81	0.77	0.72	0.73
	Extra Trees Classifier	0.77	0.81	0.77	0.73	0.70
	Extreme Gradient Boosting	0.78	0.81	0.78	0.74	0.74
	Gradient Boosting Classifier	0.77	0.80	0.77	0.72	0.72
	Random Forest Classifier	0.77	0.80	0.77	0.74	0.71
	Naive Bayes	0.74	0.78	0.74	0.71	0.72
	Quadratic Discriminant Analysis	0.62	0.79	0.62	0.74	0.65
	K Neighbors Classifier	0.75	0.73	0.75	0.70	0.71
	Ada Boost Classifier	0.72	0.73	0.72	0.69	0.70
Deep Learning (MRI)	DenseNet121	0.73	0.76	0.73	0.70	0.71
	DenseNet169	0.73	0.77	0.73	0.69	0.70
	DenseNet201	0.74	0.77	0.74	0.71	0.72
	*DenseNet264	0.76	0.77	0.76	0.72	0.73
	ResNet50	0.75	0.77	0.75	0.72	0.73
	ResNet101	0.76	0.75	0.76	0.73	0.74
	ResNet152	0.74	0.75	0.74	0.72	0.73
	ResNet200	0.74	0.75	0.74	0.72	0.73
Deep Learning (MRI + Clinical + Radiomics)	DenseNet121	0.68	0.86	0.90	0.27	0.42
	*DenseNet169	0.71	0.89	0.93	0.30	0.45
	DenseNet201	0.67	0.85	0.90	0.27	0.41
	DenseNet264	0.68	0.86	0.85	0.27	0.41
	ResNet50	0.61	0.85	0.92	0.24	0.38
	ResNet101	0.60	0.86	0.90	0.23	0.37
	ResNet152	0.60	0.83	0.90	0.23	0.37
	ResNet200	0.60	0.84	0.89	0.23	0.36

* The model selected for further analysis and comparison for each dataset.

AUC, area under the curve; csPCa, clinically significant prostate cancer.

Supplement Figure S1 Receiver operating characteristic curves of the optimal model based on different datasets in binary classification. ns, $p>0.05$; *, $p<0.05$; **, $p<0.01$.

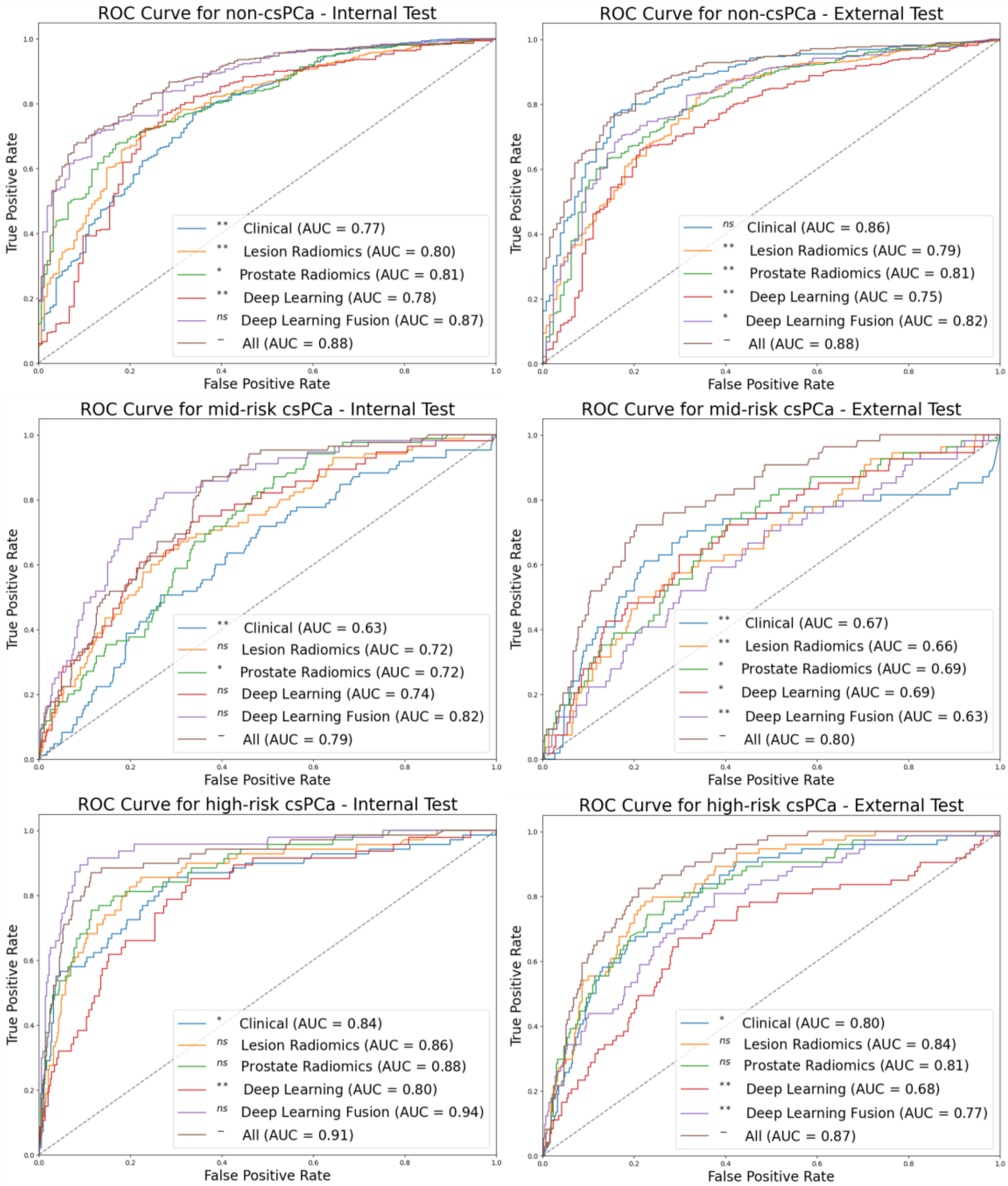

Supplement Figure S2 Performance comparison of our comprehensive framework on internal validation versus external testing. ns, p>0.05;

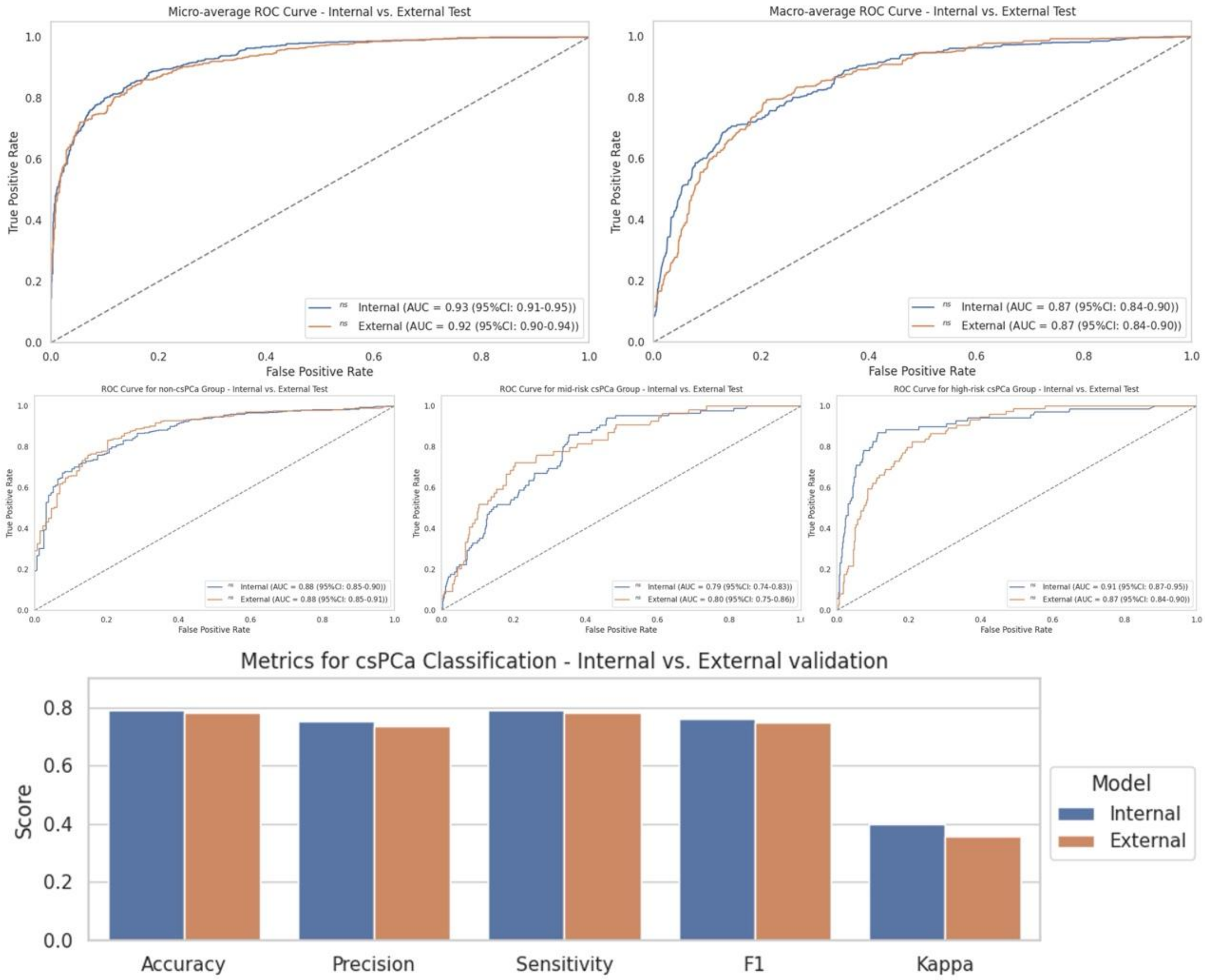

Supplement Figure S3 Receiver operating characteristic curves of our comprehensive framework stratified by PSA levels and PI-RADS 4 scores. ns, p>0.05; *, p<0.05; **, p<0.01.

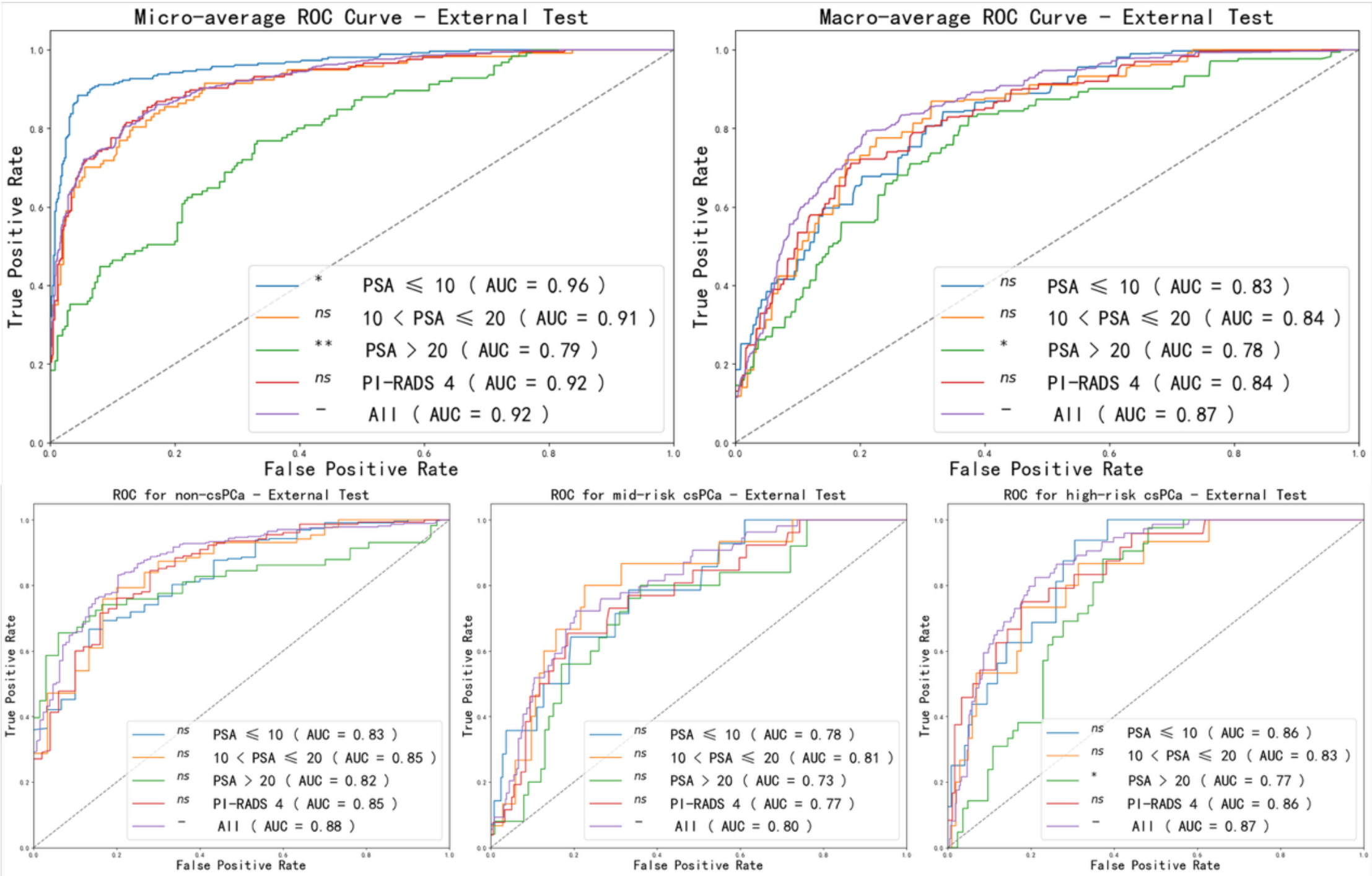

Supplement Figure 4 Receiver operating characteristic (ROC) curves comparing the diagnostic performance of the proposed model with conventional clinical variables across different PSA levels and PI-RADS 4 score. ns, $p>0.05$; *, $p<0.05$; **, $p<0.01$.

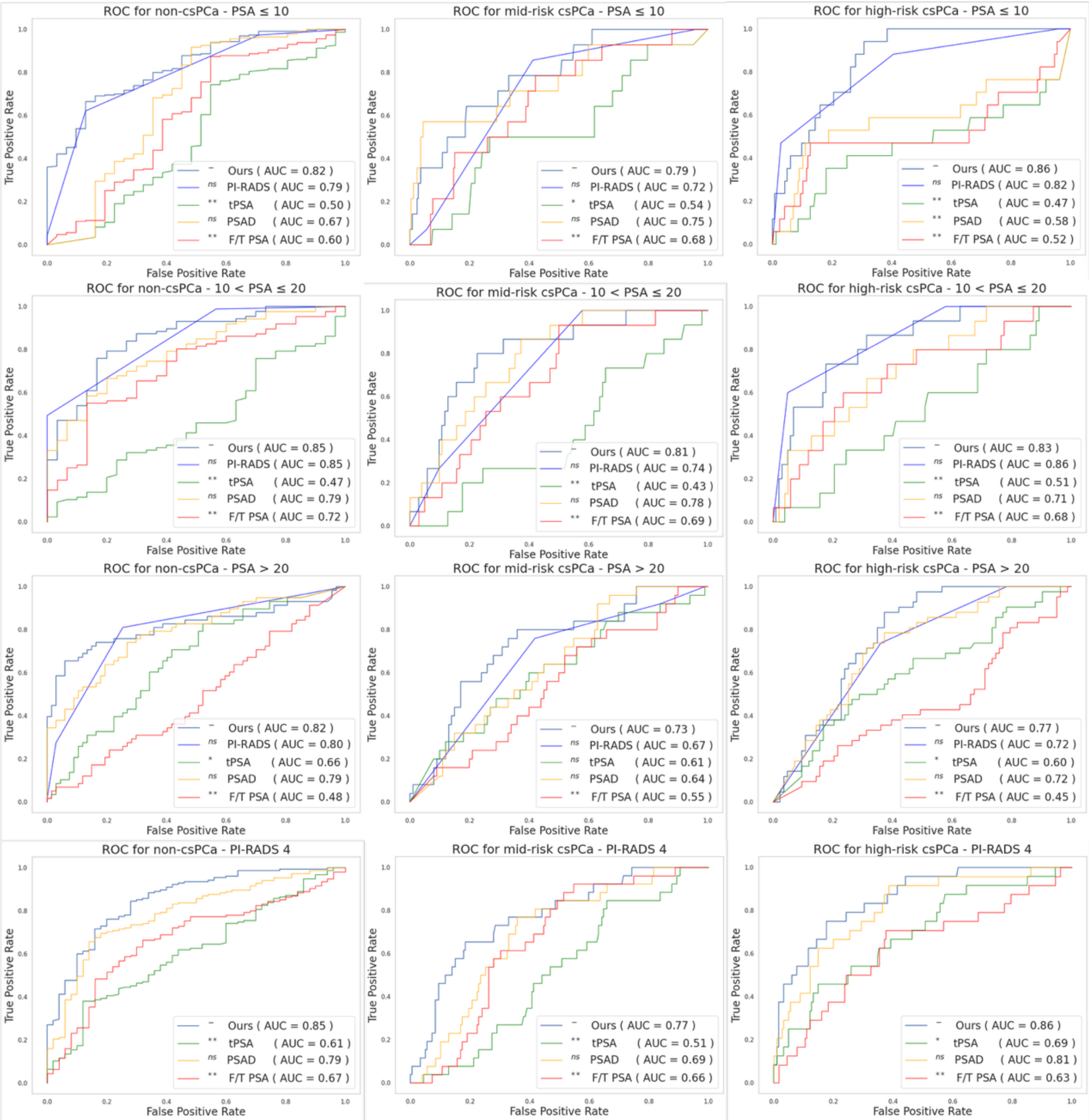

Supplement: Supplementary file 1 [file 13244_2026_2367_MOESM1_ESM.pdf]
